# Supplementary material for: Strigolactones are chemoattractants for host tropism in Orobanchaceae parasitic plants
Source: Nat Commun. 2022 Aug 15;13:4653. doi: 10.1038/s41467-022-32314-z (PMC9378612; doi:10.1038/s41467-022-32314-z)
Supplement: Supplementary file 1 — Supplementary Information [file 41467_2022_32314_MOESM1_ESM.pdf]

Strigolactones

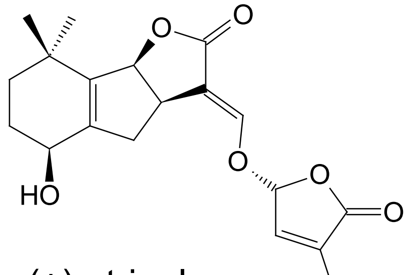

(+)-strigol

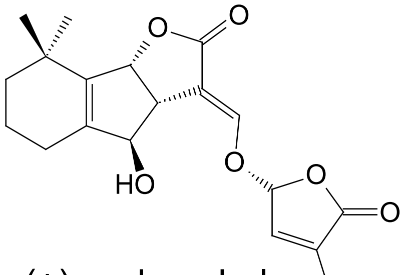

(+)-orobanchol

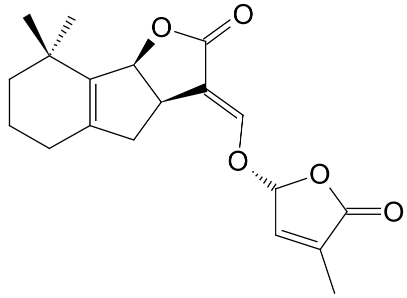

(+)-5-deoxystrigol

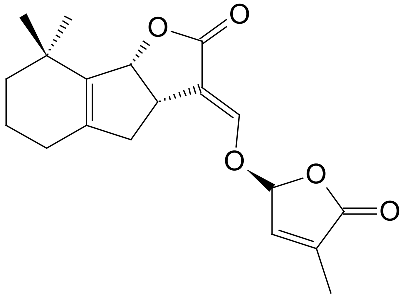

(-)-5-deoxystrigol

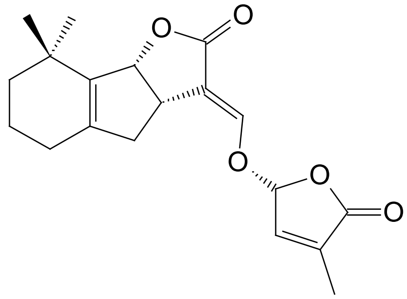

(+)-2'-*epi*-5-deoxystrigol

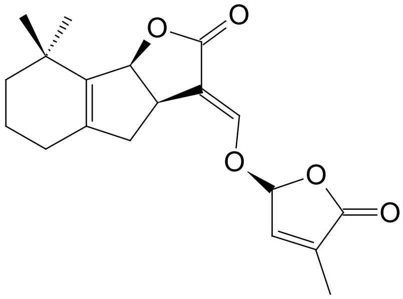

(-)-2'-*epi*-5-deoxystrigol

Synthetic analogues

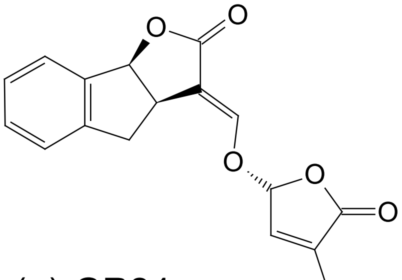

(+)-GR24

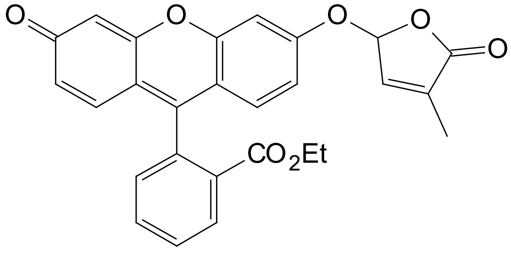

Yoshimulactone green

Supplementary Figure 1: **Chemical structure of SLs and synthetic analogues used in this study.** (+) forms are indicated for strigol, orobanchol and GR24.

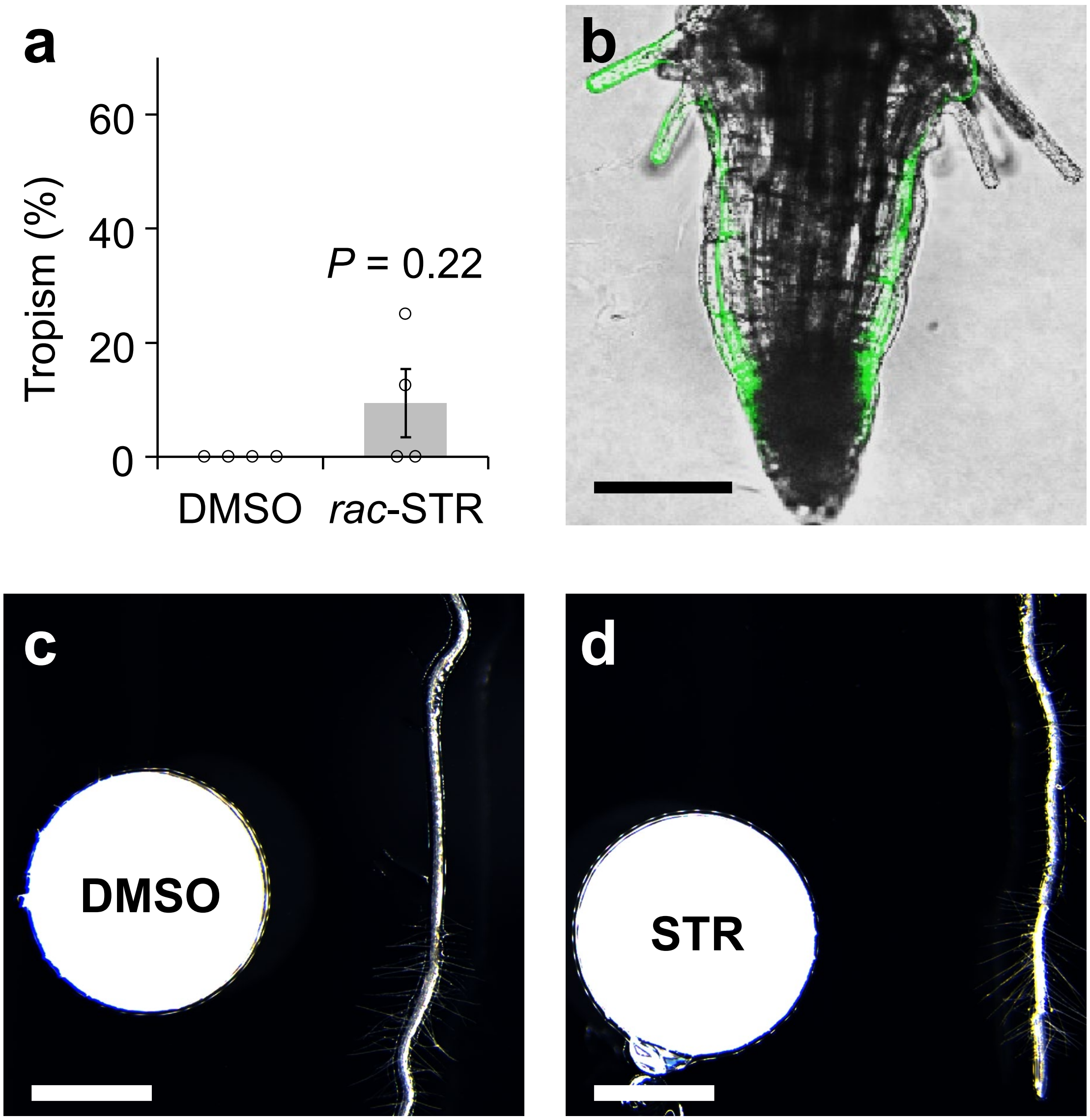

Supplementary Figure 2: **Chemotropic response to *rac*-strigol and YLG in non-parasitic plants.** **a,** Ratio of *L. philippensis* plants that showed chemotropism to 1 µM *rac*-strigol (STR) or 0.1% (v/v) DMSO. Three or four independent batches for each compound, 4-8 plants tested per batch (Mean ± SEM). Plants that stopped root growth were excluded from ratio calculation. *P*-value is indicated (Welch's *t* test, two-sided). Experiments were performed three times with similar results. **b,** A representative image of *L. philippensis* plant that showed YLG-derived fluorescence when treated with a 100 µM YLG solution. Filter paper disks were placed 3-mm to the left of the roots. Confocal photos were taken 24 hours after treatment. Bar = 100 µm. **c and d,** Representative images of *A. thaliana* plants tested for chemotropism. Photos were taken 1 day after treatment. c, 0.1% (v/v) DMSO; d, 1 µM *rac*-strigol. At least eighteen plants were tested for each chemical with similar phenotypes. Bars = 2 mm. Source data provided.

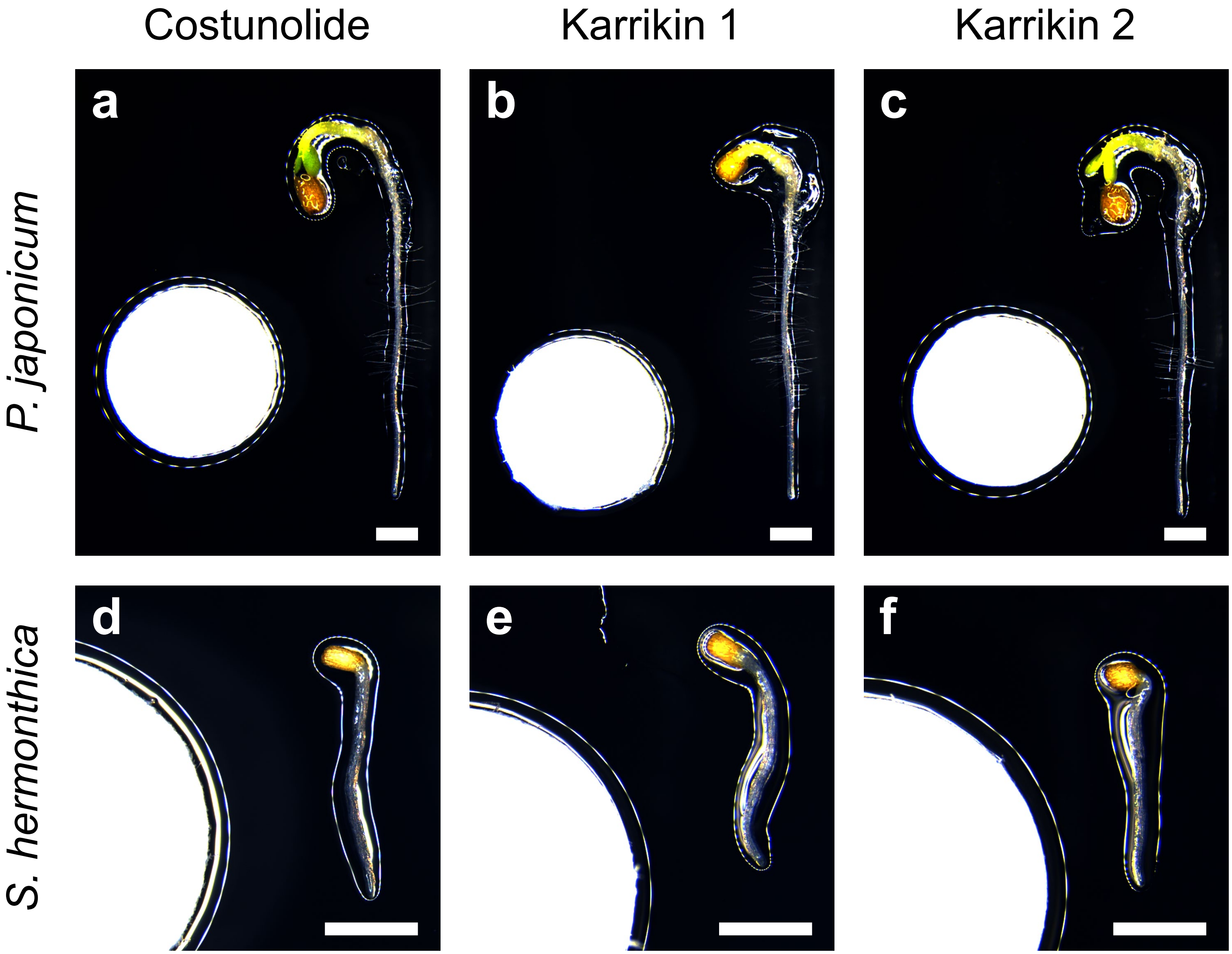

Supplementary Figure 3: **Chemotropic response to costunolide and karrikins in *P. japonicum* and *S. hermonthica*.** Representative images of *P. japonicum* (**a-c**) and *S. hermonthica* (**d-f**) plants tested for chemotropism to 1 µM chemicals in 0.1% (v/v) DMSO solution. Photos were taken 1 day after treatment. **a,d,** costunolide; **b,e,** karrikin 1; **c,f,** karrikin 2. At least seventeen *P. japonicum* plants and eleven *S. hermonthica* plants were tested for each chemical, respectively, with similar phenotypes. Bars = 1 mm.

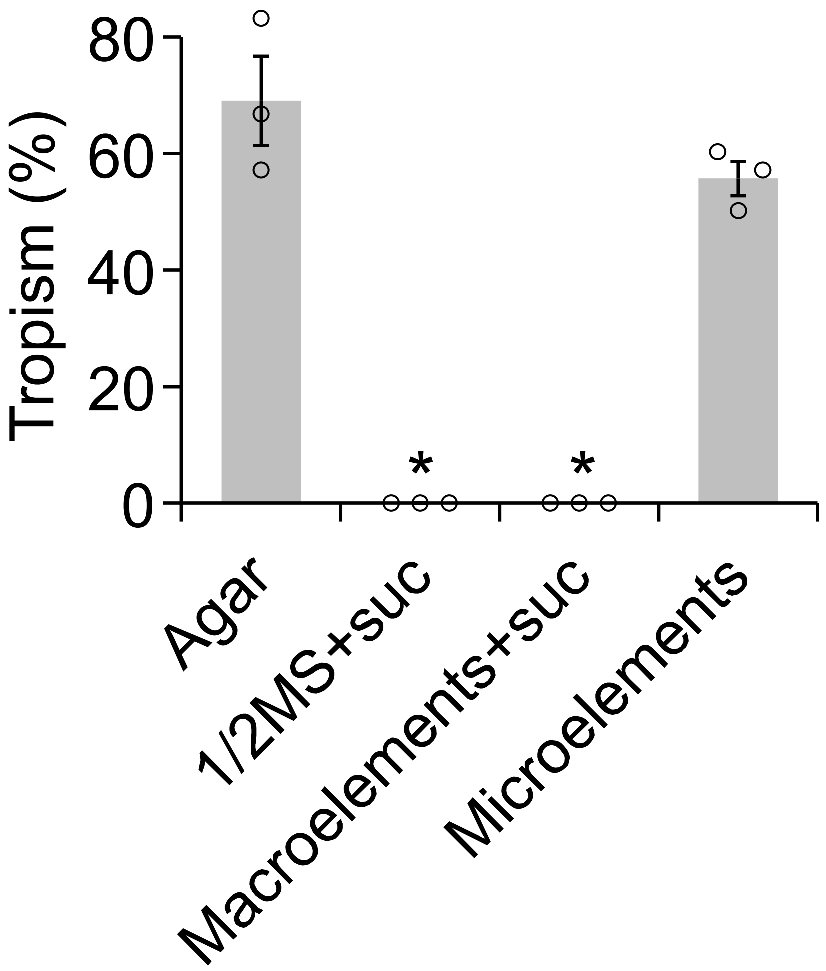

Supplementary Figure 4: **Chemotropic phenotype to *rac*-strigol on nutrient-containing media.** Ratio of *P. japonicum* plants that showed chemotropism to 1µM *rac*-strigol in each medium. Three independent batches for each compound, 6 to 8 plants tested per batch (Mean ± SEM). Plants that stopped root growth were excluded from ratio calculation. Asterisks indicate statistical significance in comparison with no nutrient condition (**P* < 0.05, Welch's *t* test, two-sided). Experiments were performed three times with similar results. Source data provided.

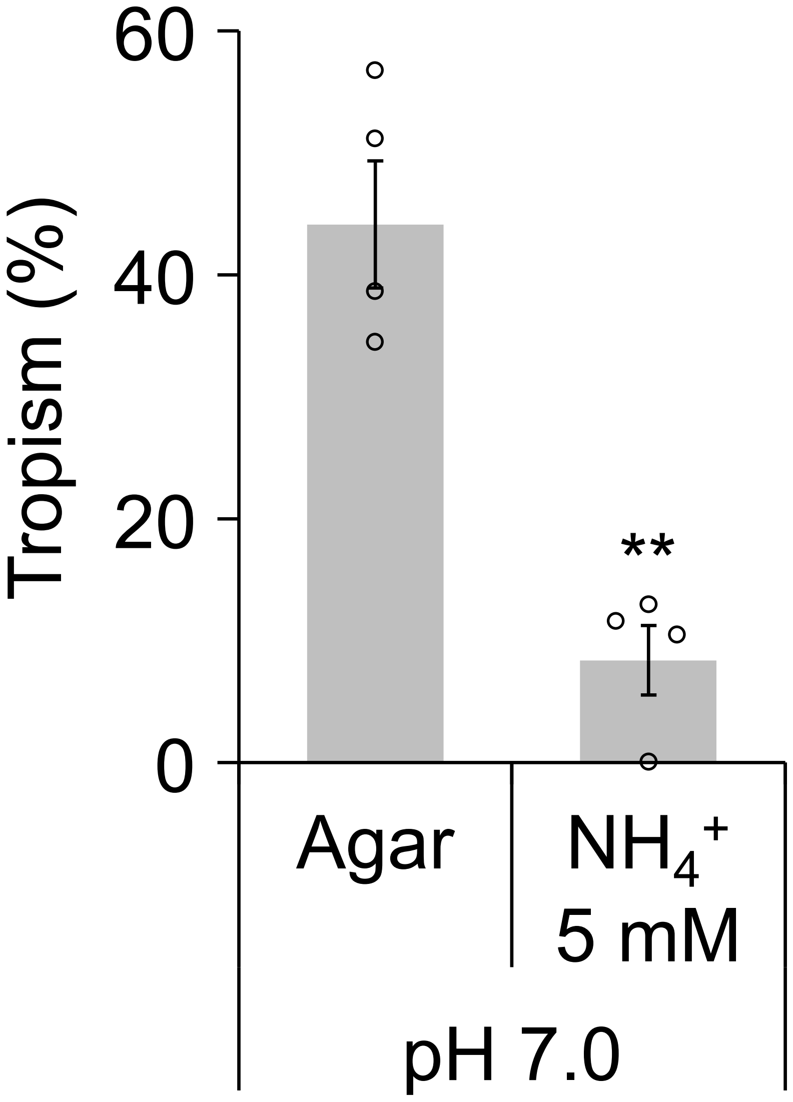

Supplementary Figure 5: **Chemotropic phenotype to *rac*-strigol on ammonium-containing media at a neutral condition.** Ratio of *P. japonicum* plants that showed chemotropism to 1µM *rac*-strigol on agar with or without 5 mM NH_4Cl at pH 7.0. Four independent batches for each medium, 7 to 10 plants tested per batch (Mean ± SEM). Plants that stopped root growth were excluded from ratio calculation. Asterisks indicate statistical significance in comparison with no nutrient condition (Welch's *t* test, $^{**}P <$ 0.01, Welch's *t* test, two-sided). Experiments were performed three times with similar results. Source data provided.

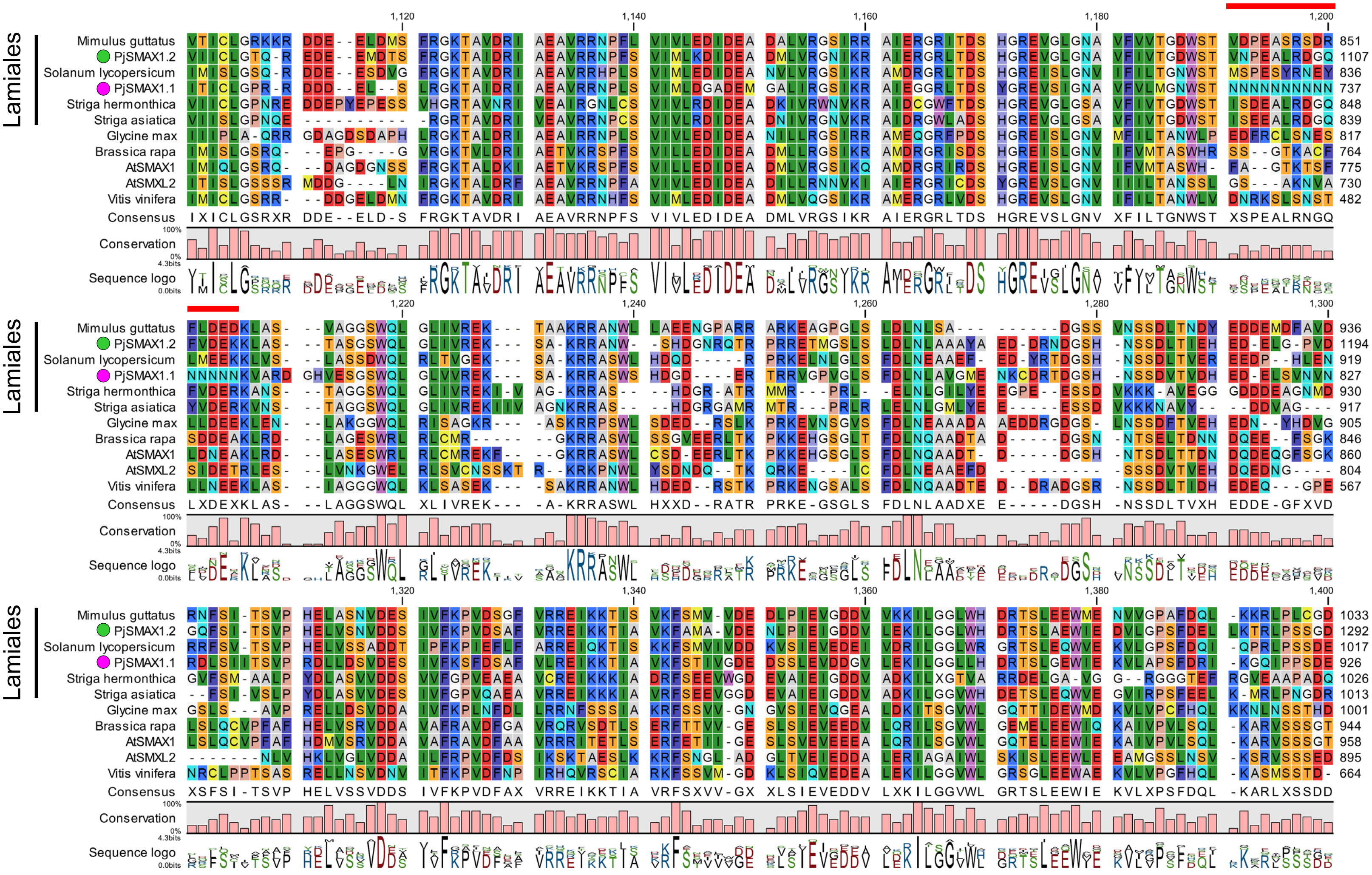

Supplementary Figure 6: **Alignment of SMAX1/SMXL2 family proteins in dicots.** Magenta and green circles indicate PjSMAX1.1 and PjSMAX1.2, repectively. Red lines indicate the region of PjSMAX1.1 with consecutive Asn, in which several amino acid sequences conserved in other SMAX1/SMXL2 proteins in Lamiales are substituted.

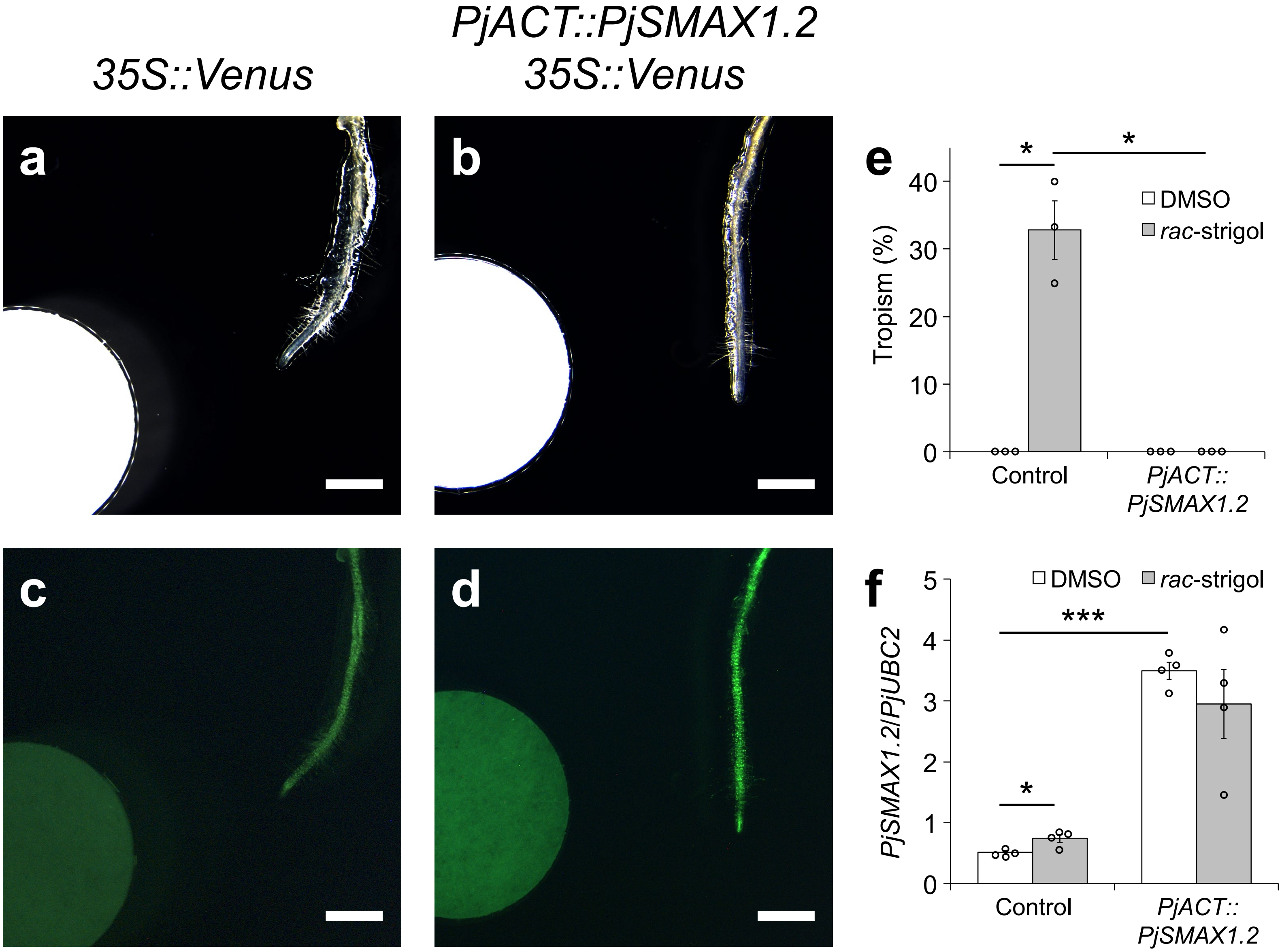

Supplementary Figure 7: **Chemotropic responses to *rac*-strigol in *PjSMAX1.2*-overexpressing plants.** **a-d**, Representative images of transgenic hairy roots treated with 1μM *rac*-strigol. (**a,b**) Bright-field images of control (**a**) and *PjSMAX1.2*-overexpressing plants (**b**). (**c,d**) Venus fluorescent images corresponding to **a** and **b**, respectively. Bars = 1 mm. **e**, Percentage of *P. japonicum* transgenic hairy roots that were chemotropic to 1 μM *rac*-strigol or 0.1% (v/v) DMSO. Three independent batches (3 to 5 plants) for each compound (Mean ± SEM). Plants that stopped root growth were excluded from the calculations. **f**, Relative expression level of *PjSMAX1.2*. Representative data are shown (4 technical replicates) using *PjUBC2* as the reference gene (Mean ± SEM). **e,f** *$P < 0.05$, ***$P < 0.001$ (Welch's *t* test, two-sided). Experiments were performed three times with similar results. Source data provided.

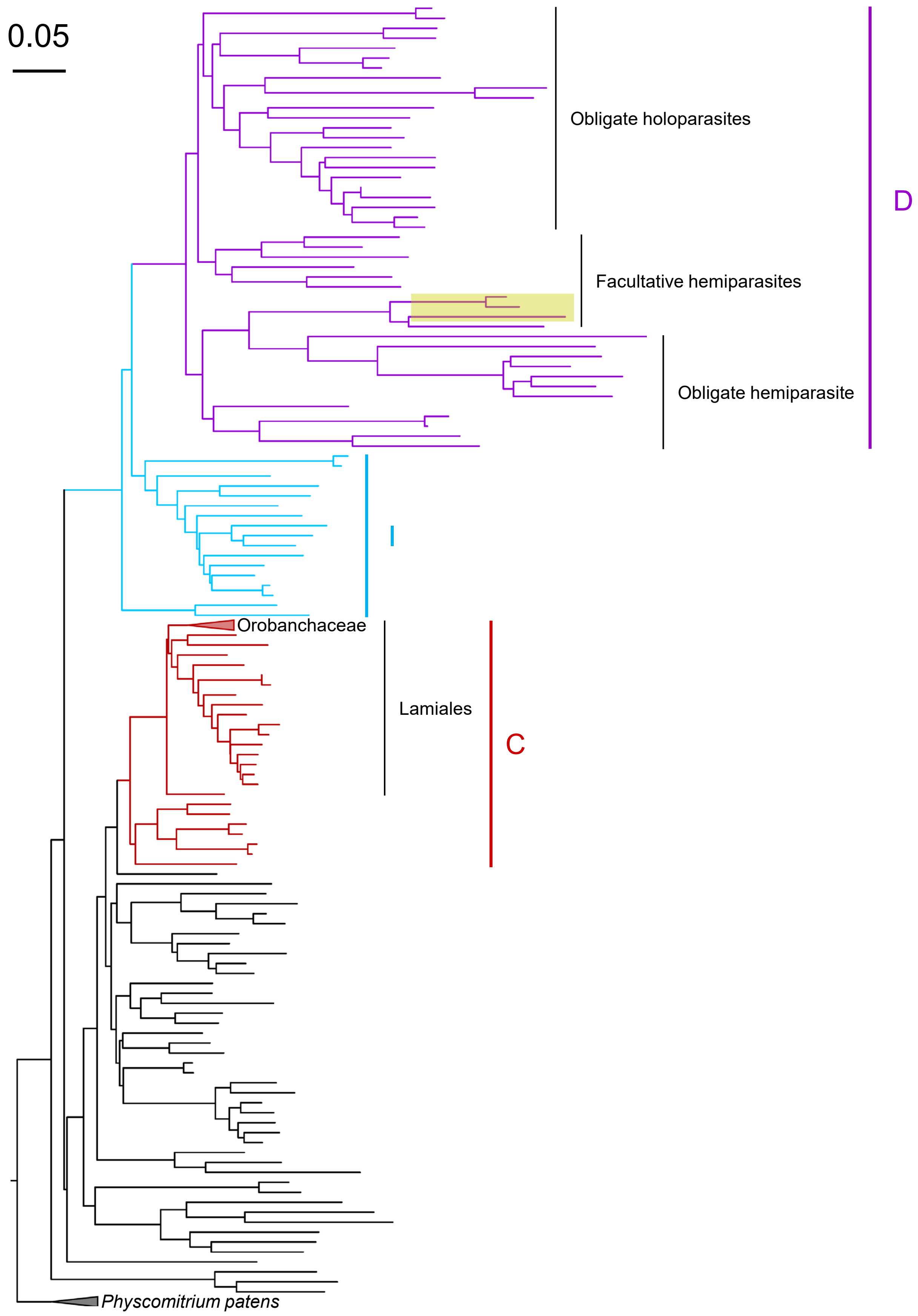

Supplementary Figure 8: **Classification of KAI2 proteins in dicots.**
Physcomitrium patens KAI2 clade was included as an outgroup. Clades in the Lamiids were colored red, blue and violet for conserved (C, KAI2c), intermediate (I, KAI2i), and divergent (D, KAI2d), respectively. Orobanchaceae clade in the KAI2c was collapsed. PjKAI2d2, PjKAI2d3, PjKAI2d3.2 were highlighted in yellow. A bar indicates substitutions per site.

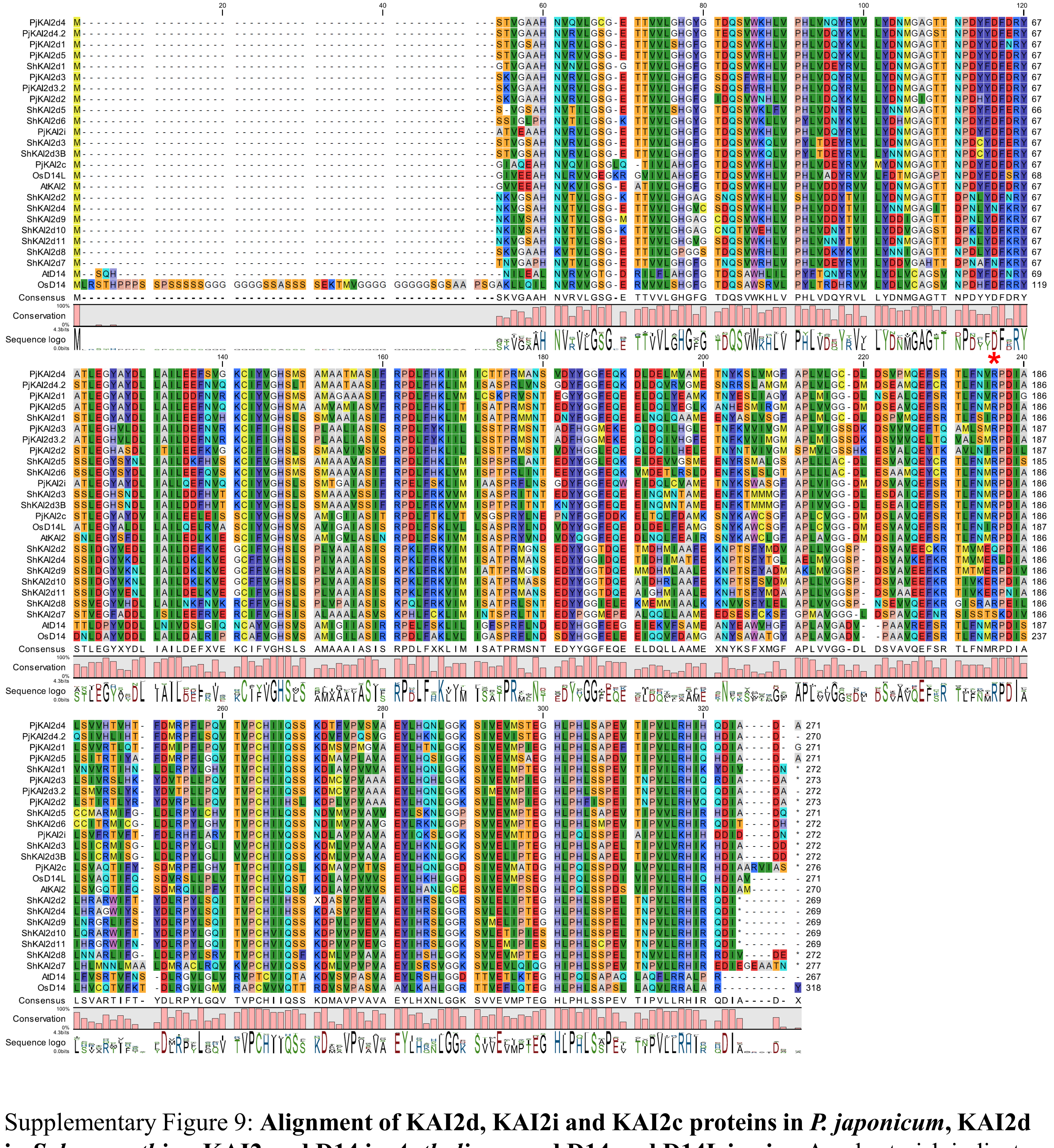

Supplementary Figure 9: **Alignment of KAI2d, KAI2i and KAI2c proteins in *P. japonicum*, KAI2d in *S. hermonthica*, KAI2 and D14 in *A. thaliana*, and D14 and D14L in rice.** A red asterisk indicates the substituted residue of PjKAI2d2 for dominant negative.

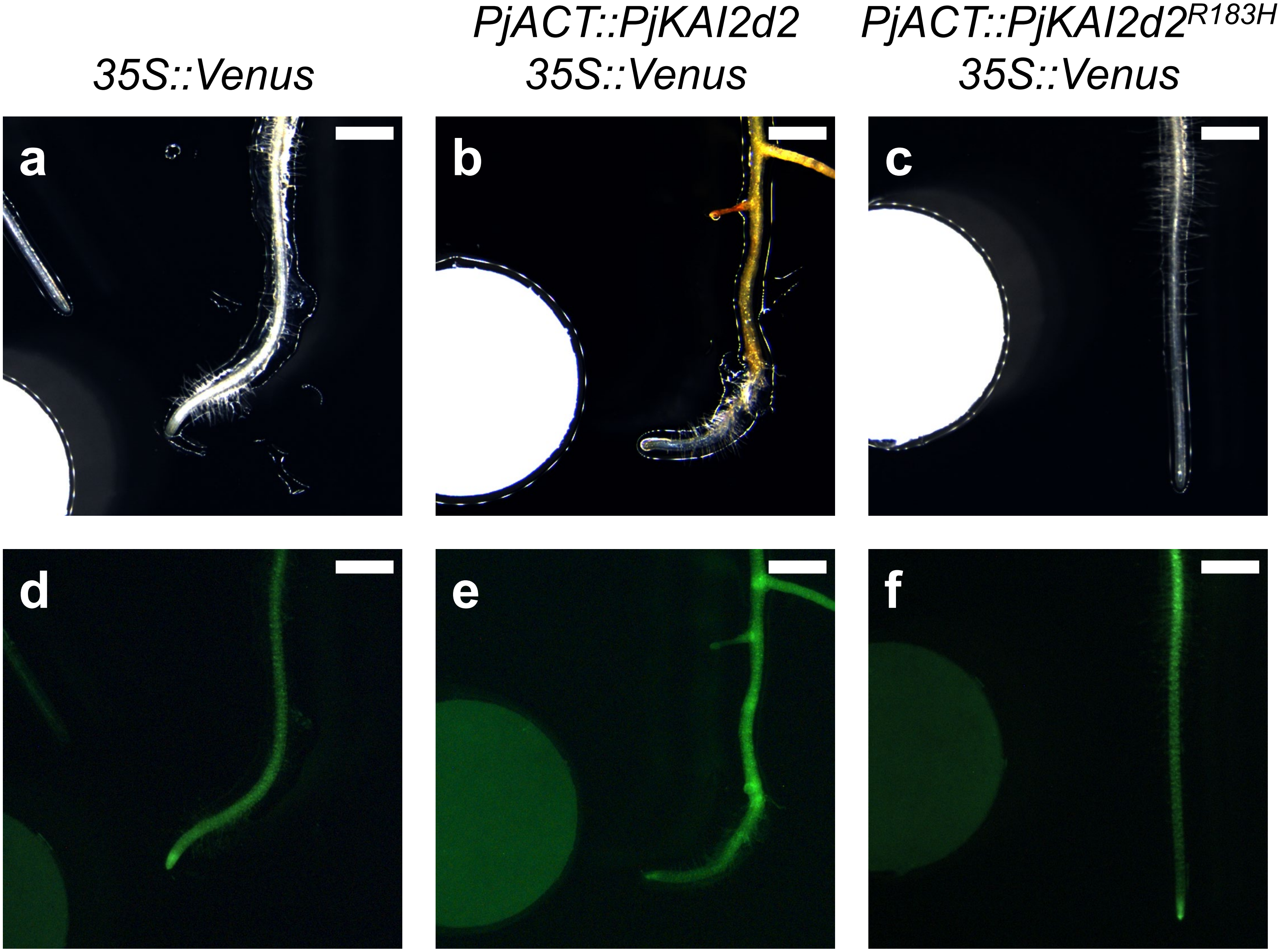

Supplementary Figure 10: **Representative images of *PjKAI2d2*-overexpressing roots treated with 1µM *rac*-strigol solution.** Photos were taken 1 day after treatment. **a-c,** Bright-field images of (**a**) Control; (**b**) *PjKAI2d2*-overexpressing plants; (**c**) *PjKAI2d2*^{R183H}-overexpressing plants. **d-f,** Venus fluorescent images corresponding to (**a-c**), respectively. Percentage of the transgenic hairy roots that were chemotropic to 1 µM *rac*-strigol was indicated in Fig. 4d. Bars = 1 mm.
