# Supplementary material for: Strigolactones are chemoattractants for host tropism in Orobanchaceae parasitic plants
Source: Nat Commun. 2022 Aug 15;13:4653. doi: 10.1038/s41467-022-32314-z (PMC9378612; doi:10.1038/s41467-022-32314-z)
Supplement: Supplementary file 3 — Description of Additional Supplementary Files [file 41467_2022_32314_MOESM3_ESM.pdf]

Description of Additional Supplementary Files

File Name: Supplementary Data 1
Description: **Chemicals used in this study.**

File Name: Supplementary Data 2
Description: **Primers used in this study.**

File Name: Supplementary Data 3
Description: **Gene IDs and accession numbers of the *P. japonicum* genes investigated in this study.**

File Name: Supplementary Movie 1
Description: **Time-lapse imaging of chemotropism to YLG in *P. japonicum*.** Filter paper disks were soaked in 1 μM YLG (left) or 0.1% (v/v) DMSO (right) and placed 5 mm from the root of a 3-day-old *P. japonicum* seedling growing on 0.7% INA agar medium (w/v). Images were taken for 18 hours at intervals of 648 seconds.

File Name: Supplementary Movie 2
Description: **Time-lapse imaging of tropism to rice in *P. japonicum*.** Root growth of *P. japonicum* was captured for 48 hours at intervals of 20 minutes after being placed between the wild-type rice (top) and *d10* (bottom) seedlings growing on 1% water agar medium (w/v). The movie was generated with 12 frames per second.
