# Supplementary material for: Strigolactones are chemoattractants for host tropism in Orobanchaceae parasitic plants
Source: Nat Commun. 2022 Aug 15;13:4653. doi: 10.1038/s41467-022-32314-z (PMC9378612; doi:10.1038/s41467-022-32314-z)
Supplement: Supplementary file 9 — Reporting Summary [file 41467_2022_32314_MOESM9_ESM.pdf]

nature portfolio

Corresponding author(s): Ken Shirasu

Last updated by author(s): Jul 25, 2022

Reporting Summary

Nature Portfolio wishes to improve the reproducibility of the work that we publish. This form provides structure for consistency and transparency in reporting. For further information on Nature Portfolio policies, see our Editorial Policies and the Editorial Policy Checklist.

Statistics

For all statistical analyses, confirm that the following items are present in the figure legend, table legend, main text, or Methods section.

n/a	Confirmed	
☐	☒	The exact sample size (*n*) for each experimental group/condition, given as a discrete number and unit of measurement
☐	☒	A statement on whether measurements were taken from distinct samples or whether the same sample was measured repeatedly
☐	☒	The statistical test(s) used AND whether they are one- or two-sided<br>*Only common tests should be described solely by name; describe more complex techniques in the Methods section.*
☒	☐	A description of all covariates tested
☐	☒	A description of any assumptions or corrections, such as tests of normality and adjustment for multiple comparisons
☐	☒	A full description of the statistical parameters including central tendency (e.g. means) or other basic estimates (e.g. regression coefficient) AND variation (e.g. standard deviation) or associated estimates of uncertainty (e.g. confidence intervals)
☐	☒	For null hypothesis testing, the test statistic (e.g. *F*, *t*, *r*) with confidence intervals, effect sizes, degrees of freedom and *P* value noted<br>*Give P values as exact values whenever suitable.*
☒	☐	For Bayesian analysis, information on the choice of priors and Markov chain Monte Carlo settings
☒	☐	For hierarchical and complex designs, identification of the appropriate level for tests and full reporting of outcomes
☒	☐	Estimates of effect sizes (e.g. Cohen's *d*, Pearson's *r*), indicating how they were calculated

Our web collection on statistics for biologists contains articles on many of the points above.

Software and code

Policy information about availability of computer code

Data collection	Fluorescent stereo microscopy, fluorescence stereo microscope (M165 FC, Leica); Time-lapse microscopy, fluorescence stereo microscope (M205 FA, Leica, for chemotropism assay) and wide zoom stereo microscope (SZX16, Olympus, for infection assay); Confocal microscopy, inverted confocal microscope (TCS SP5 II, Leica); qPCR, MxPro - Mx3000P v4.10 (Stratagene)
Data analysis	Microsoft Office Excel 2016 (Mircosoft), ImageJ 1.51j8 [for chemotropism assay], ImageJ 1.52q [for infection assay], Adobe Illustrator CS6 (Adobe), CLC Main Workbench ver. 8.0 (Qiagen), trimAL v1.2, GraphPad Prism version 8.1 (GraphPad Software), iTOL v6 (https://itol.embl.de/)

For manuscripts utilizing custom algorithms or software that are central to the research but not yet described in published literature, software must be made available to editors and reviewers. We strongly encourage code deposition in a community repository (e.g. GitHub). See the Nature Portfolio guidelines for submitting code & software for further information.

Data

Policy information about availability of data

All manuscripts must include a data availability statement. This statement should provide the following information, where applicable:
- Accession codes, unique identifiers, or web links for publicly available datasets
- A description of any restrictions on data availability
- For clinical datasets or third party data, please ensure that the statement adheres to our policy

We declare that all data in this study are available in this Article. We have provided source data for Figs. 1, 2 and 4, and Supplementary Figs. 2, 4, 5 and 7. Sequence data from this study have been deposited at the GenBank/EMBL libraries and are publicly available now. Accession numbers of the sequences are provided in Supplementary Data 3. All other data are available from the corresponding author upon requests.

Field-specific reporting

Please select the one below that is the best fit for your research. If you are not sure, read the appropriate sections before making your selection.

☒ Life sciences ☐ Behavioural & social sciences ☐ Ecological, evolutionary & environmental sciences

For a reference copy of the document with all sections, see nature.com/documents/nr-reporting-summary-flat.pdf

Life sciences study design

All studies must disclose on these points even when the disclosure is negative.

Sample size	We did not predetermine sample sizes. We set sample sizes based on our preliminary data and published papers by other researchers.
Data exclusions	No data were excluded.
Replication	We repeated all experiments independently with similar results. The numbers of repeats are provided in the figure legends.
Randomization	Plant materials were randomly selected from a larger pool of plants grown and treated under identical conditions.
Blinding	Blinding was not relevant to our study since this manuscript is on plant science.

Reporting for specific materials, systems and methods

We require information from authors about some types of materials, experimental systems and methods used in many studies. Here, indicate whether each material, system or method listed is relevant to your study. If you are not sure if a list item applies to your research, read the appropriate section before selecting a response.

Materials & experimental systems

n/a	Involved in the study	
☒	☐	Antibodies
☒	☐	Eukaryotic cell lines
☒	☐	Palaeontology and archaeology
☒	☐	Animals and other organisms
☒	☐	Human research participants
☒	☐	Clinical data
☒	☐	Dual use research of concern

Methods

n/a	Involved in the study	
☒	☐	ChIP-seq
☒	☐	Flow cytometry
☒	☐	MRI-based neuroimaging
